# Infection with male and female *Trichuris trichiura* diagnosed in a non‐endemic area

**DOI:** 10.1002/ccr3.7250

**Published:** 2023-04-23

**Authors:** Masaki Inoue, Marin Ishikawa, Sho Tanaka, Xinhan Zhang, Hiromi Okada, Takuto Miyagishima

**Affiliations:** ^1^ Department of Gastroenterology Kushiro Rosai Hospital Kushiro Japan; ^2^ Genomics Unit, Keio Cancer Center Keio University School of Medicine Tokyo Japan; ^3^ Department of Surgical Pathology Kushiro Rosai Hospital Kushiro Japan

**Keywords:** endoscopy, parasite, *Trichuris trichiura*, whipworm

## Abstract

*Trichuris trichiura* parasitizes only humans through fecal‐oral transmission. In non‐endemic areas, the frequency of endoscopic identification has been increasing due to the increasing number of immigrants from endemic countries. To prevent infection, it is important to pay attention to sanitary conditions such as soil and water sources.

## Abstract 2

Colonoscopy revealed infection with male and female Trichuris trichiura.

## 1 EXPLANATION

**FIGURE 1:**
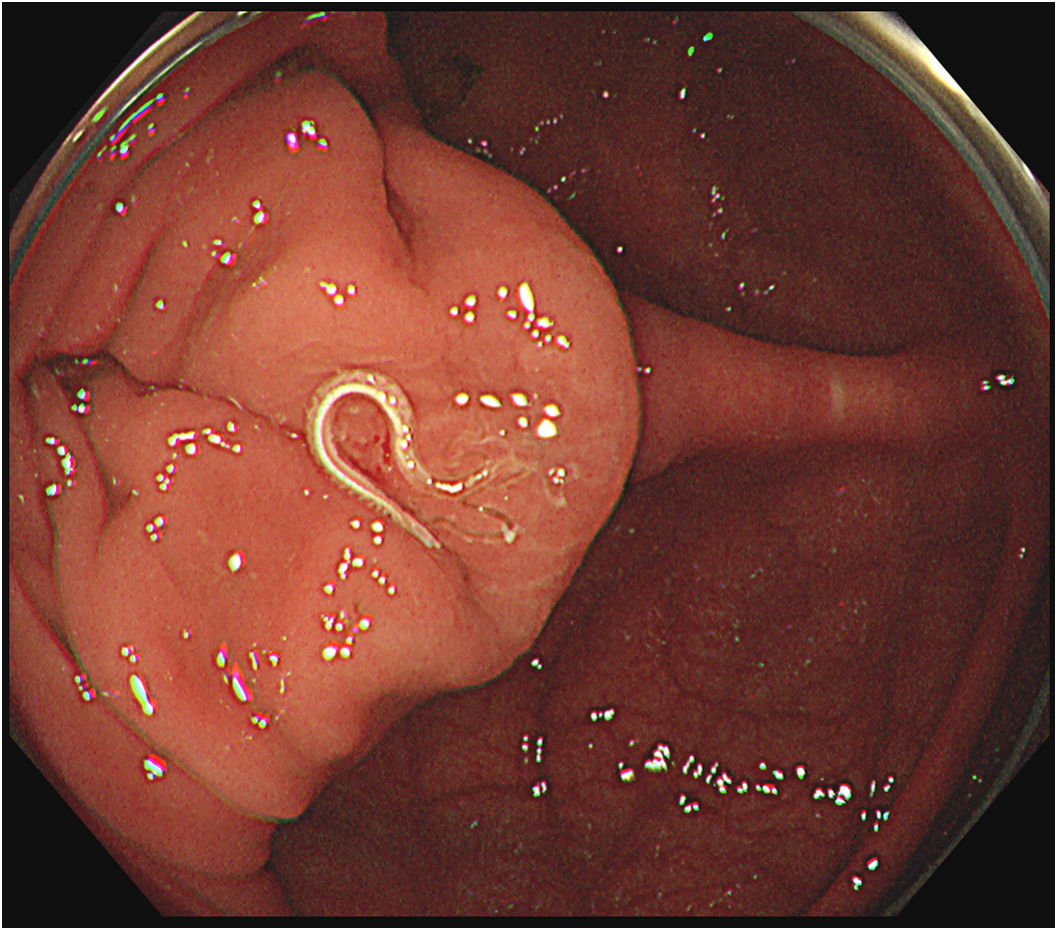
White whipworm in the cecum. Histopathological examination revealed that it was a male worm. The other two white whipworms were in the ascending colon.

**FIGURE 2:**
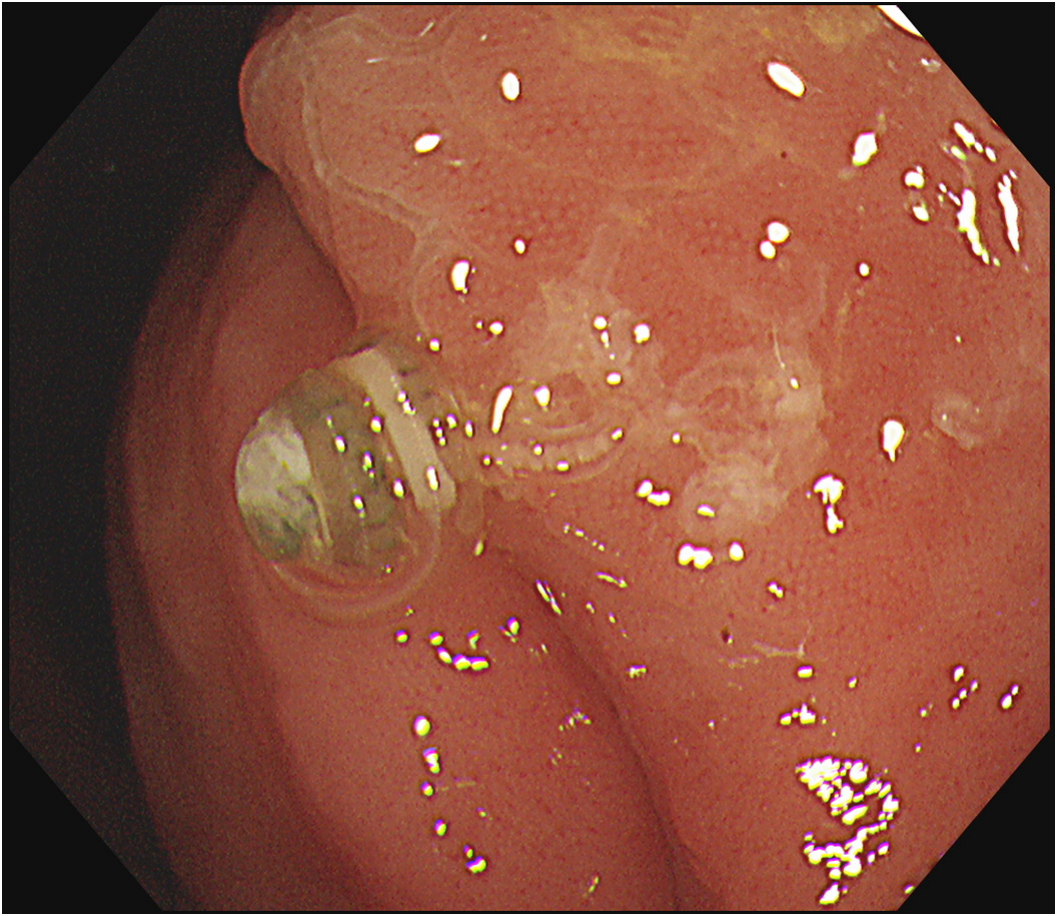
Brown whipworm in the ascending colon. Histopathological examination revealed that it was a female worm.

**FIGURE 3:**
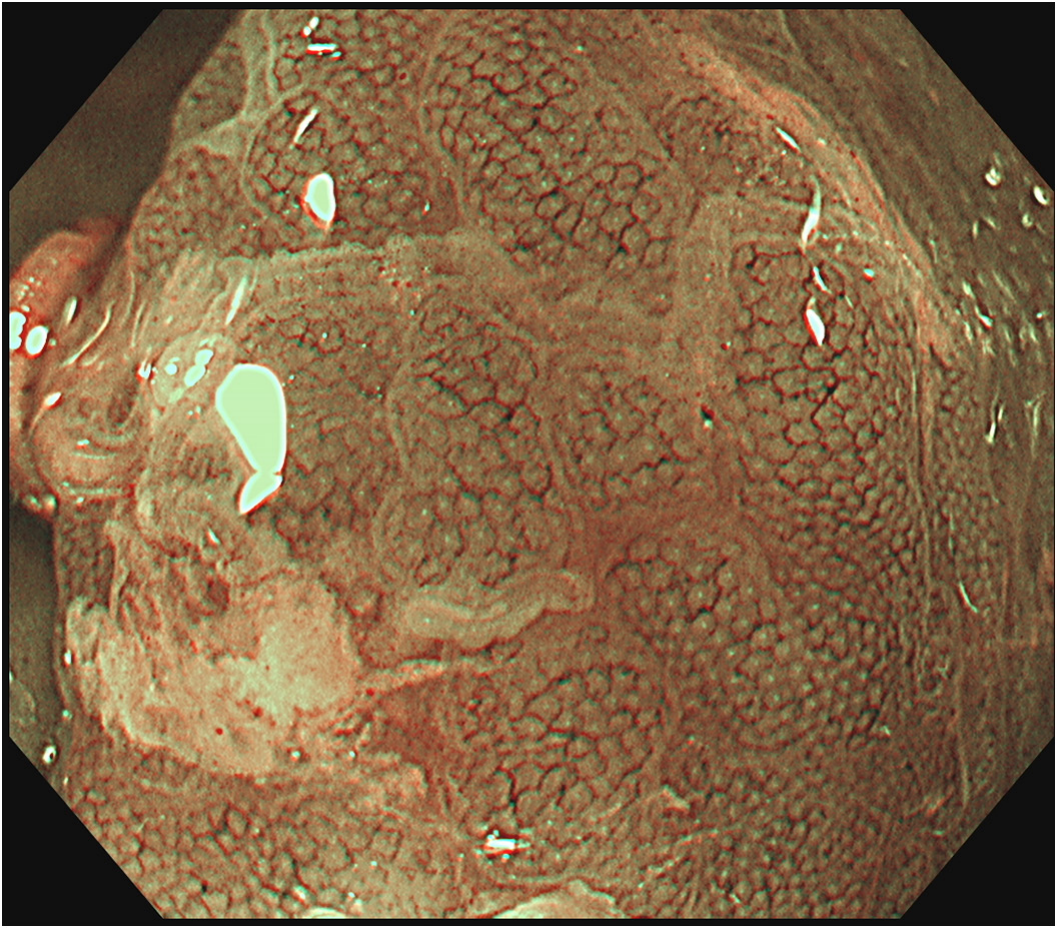
Picture of the female worm obtained by using narrow band imaging showed that its cranium was burrowing under the colonic mucosa.

**FIGURE 4:**
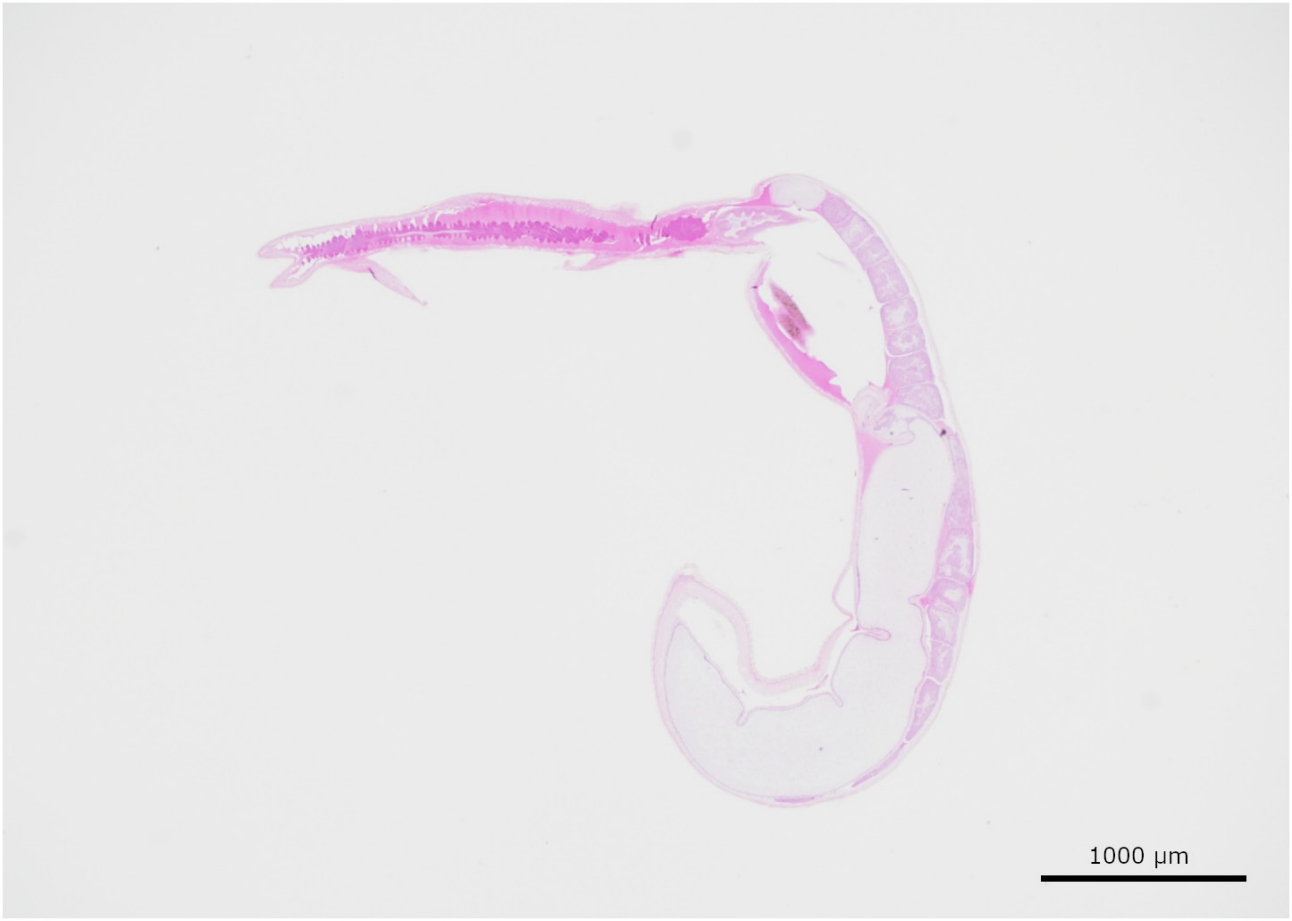
Histopathological image of a male whipworm.

**FIGURE 5:**
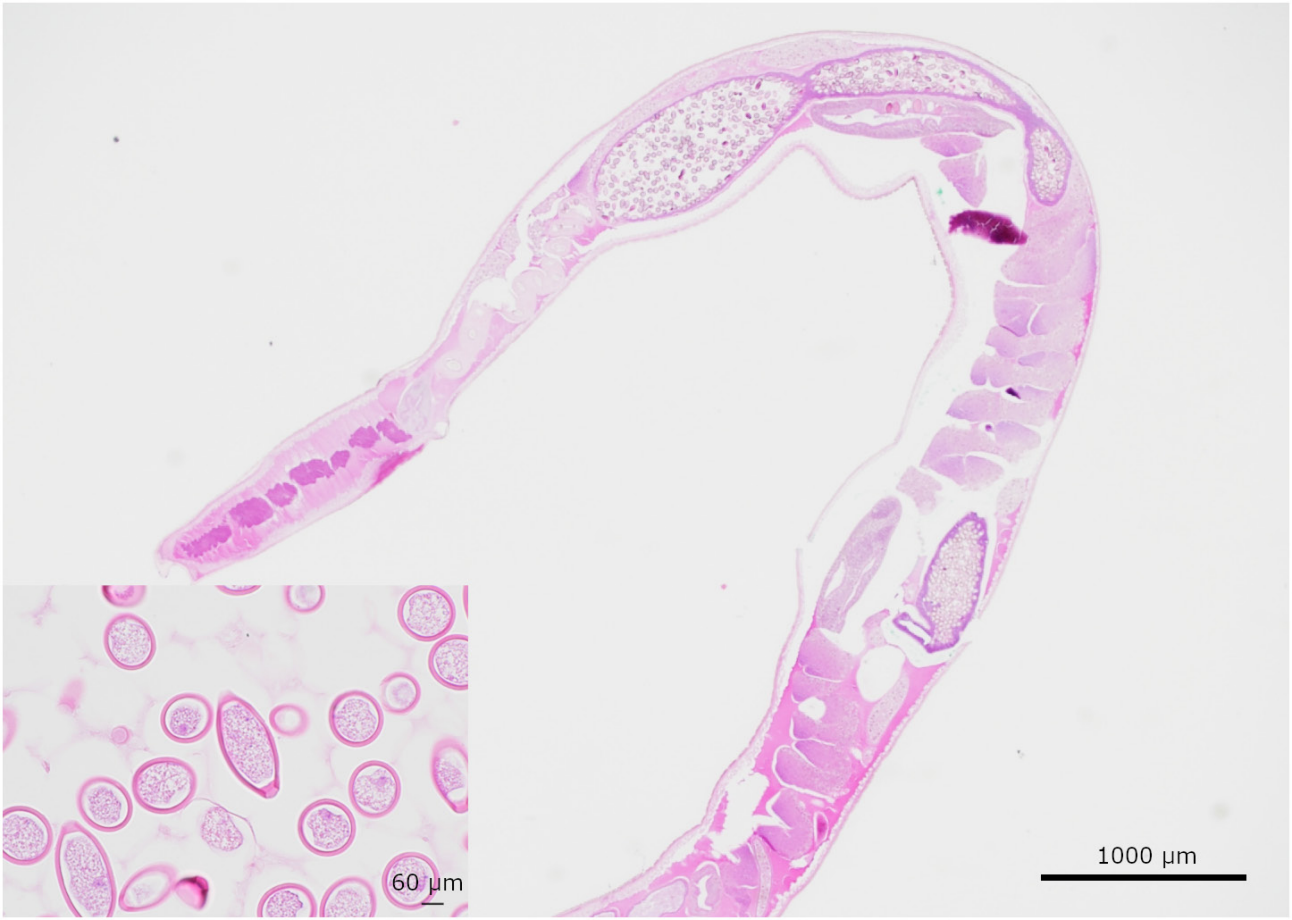
Histopathological image of the female whipworm.

A Burmese man in his 20s underwent colonoscopy at our hospital in Japan because of abdominal discomfort. He had come to Japan from Myanmar 2 years ago and had worked on a pig farm. He had had diarrhea for 5 months. Blood samples showed elevated fractions of eosinophils (white blood cells 7900/μL, eosinophils 15.6%). Colonoscopy (PCF‐H290ZI; Olympus, Tokyo, Japan) showed that there were four whipworms including one brown whipworm and three white whipworms in the cecum and ascending colon. The white whipworm was attached to the cecum mucosa (Figures 1 and 2). The brown one was detected at the ascending colon. Magnified endoscopy and narrow band imaging showed that it had a stripe pattern and that its whip‐like anterior end was burrowing into the colonic mucosa. (Figure 3). The whipworms coiled themselves up and wound slowly in response to a stimulus. We removed all of them by using biopsy forceps. Histopathological examination revealed that the brown one was a female whipworm (*Trichuris trichiura*) and the three white worms were male (Figures 4 and 5). The female had a uterus with worm eggs. A stool examination for parasites' eggs was negative. After oral administration of Mebendazole, the patient reported resolution of abdominal discomfort.

*T. trichiura* infection is prevalent in tropical regions and is non‐endemic in Japan. *T. trichiura* is thought to live for one to 8 years as an adult. Therefore, it is likely that the patient acquired the infection while in Myanmar prior to arriving in Japan. *T. trichiura* parasitizes only humans through fecal‐oral transmission. In non‐endemic areas, the frequency of endoscopic identification has been increasing due to the increasing number of immigrants from endemic countries.^1^ To prevent infection, it is important to pay attention to sanitary conditions such as soil and water sources.^2^

## AUTHOR CONTRIBUTIONS

**Masaki Inoue:** Data curation; writing – original draft. **Marin Ishikawa:** Supervision; writing – review and editing. **Sho Tanaka:** Data curation. **Xinhan Zhang:** Writing – review and editing. **Hiromi Okada:** Data curation; investigation. **Takuto Miyagishima:** Writing – review and editing.

## FUNDING INFORMATION

None.

## CONFLICT OF INTEREST STATEMENT

We have no financial relationships to disclose.

## CONSENT

Written informed consent was obtained from the patient to publish this report in accordance with the journal's patient consent policy.

## Data Availability

Data available on request due to privacy/ethical restrictions.
